# Seroprevalence and risk factors of bluetongue virus in sheep of Chattogram, Bangladesh

**DOI:** 10.14202/vetworld.2022.1589-1594

**Published:** 2022-06-30

**Authors:** Tahura Khanam Munmun, Shariful Islam, Shafayat Zamil, Md. Ashiqur Rahman, Josefina Abedin, Abdul Ahad, Ariful Islam

**Affiliations:** 1 Department of Microbiology and Veterinary Public Health, Chattogram Veterinary and Animal Sciences University, Zakir Hossain Road, Khulshi, Chattogram-4225, Bangladesh; 2 Institute of Epidemiology, Disease Control, and Research, Mohakhali, Dhaka-1212, Bangladesh; 3 EcoHealth Alliance, New York, NY, USA; 4 Centre for Integrative Ecology, School of Life and Environmental Science, Deakin University, Victoria, Australia

**Keywords:** Bangladesh, bluetongue virus, risk factors, seroprevalence, sheep

## Abstract

**Background and Aim:**

Bluetongue (BT) is a non-contagious, infectious disease of wild and domestic ruminant animals caused by the BT virus (BTV). Bangladesh having a border with a BTV-endemic country, India and a substantial number of susceptible animals. Therefore, this study aimed to estimate BTV seroprevalence and potential risk factors.

**Materials and Methods:**

We collected 150 serum samples from indigenous sheep from Chattogram, Bangladesh. We screened the serum samples using a competitive enzyme-linked immunosorbent assay for detecting BTV-specific immunoglobulin.

**Results:**

We detected antibodies against BTV in 39.3% (59/150; 95% confidence interval: 31.5–47.6) of all sampled sheep. Factors like sampling site, sheep rearing location, rearing sheep with other farm species, and body condition score had a significant (p < 0.05) influence on the seroprevalence of BTV.

**Conclusion:**

The findings show that indigenous sheep have a higher BTV seroprevalence, necessitating sustained surveillance for early diagnosis and a better understanding of virus epidemiology in Bangladesh.

## Introduction

Bluetongue (BT) is a non-contagious, vector-borne infectious disease. The causative agent, BT virus (BTV), is non-enveloped with a segmented, double-stranded RNA genome that belongs to the genus Orbivirus and family Reoviridae. Both wild and domestic ruminants such as sheep, goats, cattle, buffaloes, deer, and other Artiodactyla are vertebrate hosts of BTV [1]. *Culicoides* spp. (Diptera: Ceratopogonidae) [2] are blood-feeding insect vectors that can transmit the virus from infected viremic animals to susceptible ruminants [3]. Cattle and goats are the most common vertebrate hosts of the virus, but they generally remain asymptomatic [4]. However, sheep and deer are more likely to exhibit clinical symptoms after being infected. They sometimes result in severe systemic disorders with moderate to high mortality rates [4].

The Office International des Epizooties has classified BT as a “notifiable” illness because of its severe consequences for animal health and the economy [5]. Except for Antarctica, BTV has been found on every continent [1]. Only in areas where continuous series of virus infection cycles in vector and vertebrate host are maintained is BTV enzootic. BT has historically been found in temperate and tropical regions of the world, approximately between latitudes of 50° N and 35° S [6]. This area corresponds to the distribution of specific species of culicoides midges [7]. Environmental conditions and genetic factors of the virus, host, and vector are the primary determinants of BT activity within the vector and its ecosystem. However, the virus and vector relationship is still not well understood [8].

BTV seroprevalence has been reported from several countries within, or in the vicinity of, greater Asia, 20.3% in Tibetan sheep of China [9], 34.1% in indigenous sheep of Northwestern Ethiopia [10], 33.75–35.9% in sheep in Iran [11–14], 28.6% in small ruminants of India [15], 23% in domestic livestock of Kazakhstan [16], 39.47% in goats of Iraq [17], and 29.5% in small ruminants in Turkey [18].

A study published in 2014 from Northern Kerala, India, stated 16% seroprevalence of BTV in sheep [19], whereas conversely, in Pakistan, the seroprevalence was 56.6% in sheep [20]. Bangladesh shares its borders with India, which has long recognized BT as an endemic disease. Pakistan is also another neighboring country of Bangladesh. There are widespread movements of livestock and culicoides midges between India and Bangladesh [21–23]. Considering the high seroprevalence in neighboring countries and trans-humanism, and porous borders, it would be expected that Bangladesh also experiences BTV infections.

The status of BT and vectors involved in pathogen transmission has not been well investigated in Bangladesh, despite the high number of potential animal hosts. BT is likely misdiagnosed with other clinically similar prevalent ruminant diseases such as peste des petits ruminants (PPR) and foot rot [24] being suspected. The monsoon season (April–July) is advantageous for a rise in the abundance of various insect vectors (including culicoides) and infectious diseases. The large population of susceptible animals and favorable climatic conditions made Bangladesh a suitable place for a study of the endemicity of BT.

Thus, this study was carried out to estimate the seroprevalence of BTV in sheep of Chattogram, Bangladesh, and potential risk factors.

## Materials and Methods

### Ethical approval

The study was approved by the Ethics Committee of the Chattogram Veterinary and Animal Sciences University (CVASU) (protocol number CVASU/Dir/(R&E) EC/2015/927).

### Study design, period, and location

**Figure-1:**
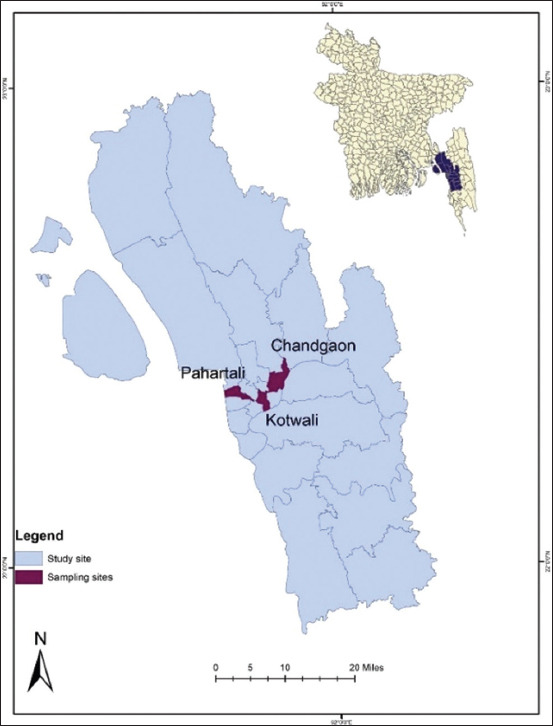
Sheep serum samples collection site in Bangladesh. [Source: DIVA-GIS (https://www.diva-gis.org/gdata].

We conducted a cross-sectional study from January to June 2018. We selected three sub-districts based on the relative abundance of sheep in each region (Figure-1). We collected 150 samples (46 from Kotwali, 94 from Pahartoli, and 10 from Chandgaon) from all the animals present on the farms of those areas at that time.

### Data collection

In Bangladesh, the farmers mostly rear the indigenous sheep, as they can easily adapt to the local environment and can be fed on low-quality feed with minimum care. Sheep are usually reared in semi-extensive housing systems along with other species of animals [23]. In all selected herds studied, an interactive pre-tested structured questionnaire was administered with the primary objective of elucidating the multifactorial background of the disease. The questionnaire included individual risk factors attributes-age (>2years and <2years) [22], sex and body condition score (BCS) (emaciated = 1; thin = 2; average = 3; fatty = 4; and obese cows = 5) [25]. The management risk factors attributes include herd size (<10 animal, 11–20 animal, 21-maximum animal), opportunity of grazing (No, rotational), vector control measures practiced on farms, source of animals in the farms (own farm, purchased from other farms or local market), is there any clinical symptoms such as fever, coughing, sneezing, and diarrhea, presence of other animal species in the herd (goat, camels) and farmyard (sheep kept in-doors or out-doors), vaccination against PPR, sheep pox, hemorrhagic septicemia (HS), and black quarter (BQ).

### Sample collection and processing

From the jugular vein of each sheep, about 4–5 mL of blood was collected aseptically. To obtain serum, the blood samples were kept at room temperature (~25°C) for 1–2 h and then centrifuged at 402× *g* force. A clear straw-colored serum was observed up around the clotted clump, which was put into a marked 1.5 mL sterile microtube (Eppendorf, Germany) and refrigerated at −20°C and sent to the Poultry Research and Training Center, CVASU for laboratory analysis.

### Laboratory analysis

According to the manufacturer’s procedures, BTV group-specific antibodies were detected using the BT Antibody Test Kit; competitive enzyme-linked immunosorbent assay (cELISA) (IDvet, 310, rue Louis Pasteur, 34790 Grabels-France). The optical density (OD) of the plate was measured using a spectrophotometer (Mindray Mr-96A, Guangdong, China) at 450 nm. If the mean value of the negative control OD (ODNC) is >0.7 and the ratios of the mean values of the positive control OD and ODNC are <0.3, the test is considered valid. We calculated the competition percentage for each sample, S/N % = OD sample/OD NC × 100. Samples presenting an S/N% ≥40% were considered negative, and <40% were considered positive.

### Statistical analysis

We entered data in MS Excel and analyzed it in STATA-13 (StataCorp, 4905, Lakeway Drive, College Station, Texas 77845, USA). We categorized the BCS as follows: poor for BCS 1 and BCS 2; fair for BCS 3; and good for BCS 4 and BCS 5. Associations between seroprevalence and potential risk factors were tested by Fisher’s exact. Risk factors were considered statistically significant if their p < 0.05. Significant risk factors were then forwarded to multiple logistic regression analysis. The validity of the multivariate logistic regression model was tested by the goodness of fit test and by the receiver operating curve (ROC).

## Results

**Figure-2:**
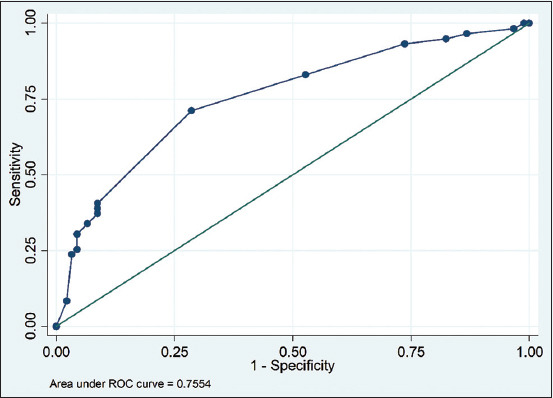
Plot of true positive percentage versus false-positive percentage for a receiver operating characteristic curve of the final multivariable logistic regression analysis of bluetongue virus in sheep of Bangladesh.

We detected BTV-specific antibodies in 39.3% (n = 59; 95% confidence interval-CI: 31.5–47.6) of all sheep samples. In univariate analysis, factors such as sampling site, age, herd size, rearing sheep with other species, vector control, and vaccination against PPR had a significant (p < 0.2) influence on the seroprevalence of BTV (Table-1). No sheep were vaccinated against Sheep pox, HS, and BQ. In the final multivariable logistic regression analysis, we found no two-way interaction between the variable with a significant difference. Sheep sampled from Chandgaon (OR: 26.79; 95% CI: 01.10–653.93) and Pahartoli (OR: 13.51; 95% CI: 03.49–52.30) area and herd size of about <10 animals (OR: 4.94; 95% CI: 01.38–17.64) had significantly higher (p ≤ 0.05) odds of being seropositive for BTV (Table-2). The goodness of fit test was insignificant (p = 0.3445) for the final model. But the area under the ROC was 0.75, suggesting that our final model fitted well with the data and had a high predictive ability to differentiate seropositive and seronegative sheep (Figure-2).

**Table 1:** Potential risk factors for seroprevalence of BTV in Sheep of Chattogram, Bangladesh (n = 150).

Variables	Category	n	Positive n (%)	p-value (Fisher exact)
Sampling sites	Kotwali	46	11 (23.91)	0.002
	Pahartali	94	40 (42.55)	
	Chandgaon	10	08 (80.00)	
Age	> 2 years	52	16 (30.77)	0.160
	< 2 years	98	43 (43.88)	
Sex	Male	125	48 (38.40)	0.657
	Female	25	11 (44.00)	
Source of animal	Own farm raised	36	16 (44.44)	0.558
	Purchase from Market	114	43 (37.72)	
Herd size	< 10	24	14 (58.33)	0.096
	11–20	61	20 (32.79)	
	21-max	65	25 (38.46)	
Opportunity of grazing	Zero	125	47 (37.60)	0.374
	Rotational	25	12 (48.00)	
Other species reared with sheep on the same farm	Yes	138	57 (41.30)	0.127
	No	12	02 (16.67)	
Vector control	Yes	23	06 (26.09)	0.173
	No	127	53 (41.73)	
BCS	Good	137	56 (40.88)	0.249
	Fair	13	03 (23.08)	
Vaccination against PPR	Yes	23	06 (26.09)	0.173
	No	127	53 (41.73)	

BTV = Bluetongue virus, BCS = Body condition score, PPR = Peste des Petits Ruminants

**Table 2:** Risk factors analysis by multivariable logistic regression for bluetongue in sheep in Chattogram, Bangladesh.

Variables	Category	OR	p-value	95% CI
Sampling sites	Kotwali	1		
	Chandgaon	26.79	0.044	01.10–653.93
	Pahartoli	13.51	0.001	03.49–52.30
Age	> 2 years	1	-	
	< 2 years	2.07	0.072	01.00–04.60
Herd size	21-Max	1	-	
	11–20	3.05	0.380	00.25–36.66
	< 10	4.94	0.014	01.38–17.64
Other species reared with sheep in same farm	No	1	-	
	Yes	15.01	00.14	0.41–550.35
Vector control	Yes	1		
	No	1.2	0.90	0.07–21.70

OR = Odds ratio

## Discussion

The study confirmed the presence of BTV antibodies in sheep from Bangladesh and identified associated risk factors related to BTV infection based on the study’s aim. Several previous studies were conducted for serological diagnosis of BTV by monoclonal antibody-based cELISA targeting VP7 protein [26] from Colorado, USA [6], China [9], Ethiopia [10], etc. Hence, we used cELISA for our study to estimate the seroprevalence.

The overall seroprevalence of BTV in sheep in Chattogram was 39.3%. This is much higher than in our neighboring country India, where 16% of sheep from Kerala were positive for BTV-specific antibody [19]. However, our finding agrees with a previous report of 34.1% BTV seropositivity in indigenous sheep of Northwestern Ethiopia [10]. Another study from China detected a BTV antibody among 20.3% samples of Tibetan sheep [9]. Several studies from Iran reported BTV antibody in 33.75% to 35.9% of sheep [11–14].

The present finding is relatively lower than the seroprevalence of BTV in small ruminants previously reported by different authors in different countries, such as 41.17% in small ruminants in Southern Ethiopia [27], 46.67% in sheep in central Ethiopia [28], 78.4% in small ruminants in Grenada [29], 56.6% in sheep in Pakistan [20], and 45.7% in small ruminant in India [30]. However, the current study’s 39.3% seropositivity to BTV in sheep is higher than the prior research estimates of 6.57% seropositivity in sheep in Southeast Iran [12] and 5.70% in sheep in Algeria [31]. The difference in the seropositivity could be related to difference in the sampled species, age, sex, immune status, types of rearing, and specifically for sheep, different approaches to rearing with other species of animal and vector control measures employed around sampled animals.

We found variation in the seroprevalence of BTV among three selected sampling sites of Chattogram. Sheep from Chandgaon showed the highest positivity (80%). Malik *et al*. [20] identified sampling sites as a significant risk factor for BTV seropositivity in sheep, and also reported variation in the prevalence of BTV in different locations in Ethiopia. The varying seroprevalence of BTV in other study sites in this study may be due to differing sample sizes and geographical variations.

The results from our study demonstrated a trend in the number of seropositive animals decreasing with age, though it was ultimately not statistically significant. Moreover, BTV seroprevalence was higher among sheep aged between 1- and 2 years [19]. Young animals are generally kept indoors and well cared for by their owners, especially when avoiding insect and tick-borne infections. It was found that the younger animals started to get an infection with BTV when they were allowed into the field for grazing at the age of 6 months. Hence, it was not unlikely to get higher seropositivity in younger animals aged <2 years. They are more likely to be exposed to vectors and subsequent BTV infection in grazing areas. Sabaghan *et al*. [32] found significant differences in BTV antibodies among males and females, but we detected no significant difference between sexes for the presence of BTV antibodies in our study.

Different farms have different management tactics, which influence animal exposure to BTV. Herd size is also identified as a significant risk factor for BTV seropositivity in sheep [20]. In our study, BTV antibodies were prevalent in sheep experiencing rotational grazing. Out-door grazing greatly increases the potential for exposure to vectors compared to sheep with zero grazing.

In the present investigation, no significant difference (p > 0.05) in BTV seroprevalence was observed due to vector control and vaccination against PPR of sampled sheep. Vector control and vaccination against PPR were practiced only in a small number of animals. All these factors may influence the insignificant variation among different groups. Similarly, there was no significant difference (p < 0.05) in BTV seroprevalence by BCS of sheep and sheep reared with other species.

It is also acknowledged that sheep housed or grazed with other ruminant species such as goats, cattle, and deer, in the presence of abundant levels of culicoides midges, may have a high BTV titer with often minimum clinical manifestations [27]. These species may be a reservoir source of infection for other vulnerable animals. We found a significantly high prevalence (41.3%) of BTV in sheep that were reared with other species such as goats and cattle.

The seroprevalence of BT has been detected among sheep in different areas of Chattogram, indicating exposure to infection may be more widely distributed over the country. No clinical case of BT was observed among animals during sampling. To date, no cases of BT have been reported in Bangladesh. The absence of clinical disease may be due to a high degree of innate immunity in the local breed of sheep [33]. This phenomenon could be altered if more virulent strains are introduced, or host resistance is lower by crossbreeding.

As the sheep population in the Chattogram district was smaller than that of other districts, the samples collected from the district were proportionately fewer. Moreover, we did not perform a serum neutralization test with the serosurvey. cELISA is a test with high sensitivity and specificity though there is a chance of cross-reaction with similar viruses. In the future, a longitudinal study should be done to isolate the virus from sheep and identify the serotype to assist with developing improved control measures against BTV infection in sheep in Bangladesh.

## Conclusion

This present study detected BTV antibodies in sheep and identified potential risk factors associated with transmission of BTV and control of the disease. BTV-specific antibodies were widespread in the study areas, though active disease outbreak/incidence has not been reported in sheep so far. Further studies should be conducted to identify the virus and determine the specific serotypes circulating in this country. This will enable a more detailed epidemiology of the disease to become available, allowing more suitable control measures to be introduced into Bangladesh.

## Authors’ Contributions

AI: Conceptualization. TKM, SI, SZ, MAR, and JA: Methodology. TKM, SI, and SZ: Software. AI, AA, and JA: Validation. AI, TKM, and SI: Formal analysis. AI and AA: Investigation. AI and AA: Resources. TKM and MAR: Data curation. TKM, SI, and AI: Drafted the manuscript. TKM, SI, SZ, AI, and AA: Reviewed and edited the manuscript. SI and SZ: Visualization. AI and AA: Supervision. AI: Project administration. All authors have read and approved the final manuscript.
